# Coherent optical spectroscopy in a biological semiconductor quantum dot-DNA hybrid system

**DOI:** 10.1186/1556-276X-7-133

**Published:** 2012-02-16

**Authors:** Jin-Jin Li, Ka-Di Zhu

**Affiliations:** 1 Key Laboratory of Artificial Structures and Quantum Control (Ministry of Education), Department of Physics, Shanghai Jiao Tong University, 800 Dong Chuan Road, Shanghai 200240, China

## Abstract

We theoretically investigate coherent optical spectroscopy of a biological semiconductor quantum dot (QD) coupled to DNA molecules. Coupling with DNAs, the linear optical responses of the peptide QDs will be enhanced significantly in the simultaneous presence of two optical fields. Based on this technique, we propose a scheme to measure the vibrational frequency of DNA and the coupling strength between peptide QD and DNA in all-optical domain. Distinct with metallic quantum dot, biological QD is non-toxic and pollution-free to environment, which will contribute to clinical medicine experiments. This article leads people to know more about the optical behaviors of DNAs-quantum dot system, with the currently popular pump-probe technique.

## 1 Introduction

Rapid and highly sensitive detection of DNA molecules contributes to ultrasensitive and automated biological assays such as sensing, imaging, immunoassay, and other diagnostics applications [1-3]. Conventional approaches have focused on inorganic/organic hybrid DNA biomedicine sensors for biological labels, cell tracking, and monitoring response to therapeutic agents [4-6]. Among numerical hybrid components, the unique size-dependent, narrow, symmetric, bright, and stable fluorescence of quantum dots (QDs) have made themselves powerful tools for investigating a wide range of biological problems [7]. This is a difficult task with standard fluorophores because their relatively narrow excitation and broad emission spectra often result in spectra overlap. Besides, the optical behaviors of quantum dots are typically unaffected while they are conjugating to bio-molecules, which make them highly stable and bright probes, especially suitable for photon-limited in vivo studies and continuous tracking experiments over extended time periods [7]. Recently, the coherent optical spectroscopy of a strongly driven quantum dot has been experimentally investigated by Xu et al. [8,9]. They have shown that, like single atom two- and three-level quantum systems, single QD can also exhibit interference phenomena including Autler-Townes splitting and gain without population inversion when driven simultaneously by two optical fields. In this case, researchers are indulged in quantum dots and DNA conjugates to study biological activities and medical diagnosis [10,11], which have applications in biomolecule targets exploitation [12,13]. But for the research of coherent optical spectrum in such coupled DNAs-QD, no study has ever been undertaken, neither in experiment nor in theory.

Furthermore, there is another question. The metallic quantum dots used in biological assays always have toxicity, which may limit the capabilities of biomedicine assays and bring in some unnecessary troubles. So the search for cadmium-free quantum dots has therefore becomes another major research area. Most recently, Amdursky et al. [14,15] have experimentally demonstrated that the peptide quantum dots represent one of the simplest forms of quantum dot and the most important feature of these quantum dots is the nontoxicity to the environment and to human body. These quantum dots will become new labeling materials in biological and biomedical assays. However, the coherent optical properties of such QDs coupled to DNAs are still lacking.

In the present study, we theoretically investigate the coherent optical spectroscopy for a peptide quantum dot (QD) coupled to DNA molecules, with pump-probe technique. Recently, this two-laser technique has been realized by several groups [16-20] while investigating the optomechanical system. Here we show that this hybrid peptide QD-DNA system will become transparent due to the DNA's vibrations when applying a strong control laser. Under some conditions the output signal laser even be enhanced significantly. Furthermore, the vibrational frequency of DNA molecule and the coupling strength between peptide QD and DNA can be measured due to the absorption splitting peaks in all-optical domain.

## 2 Model and theory

**Figure 1:**
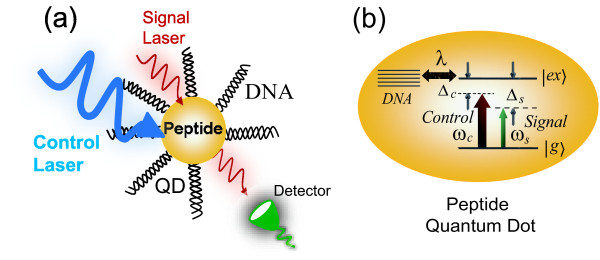
**Schematic diagram of a peptide quantum dot coupled to DNA molecules**. **(a) **A single peptide QD coupled to DNA molecules in the simultaneous presence of two optical fields. **(b) **The energy levels of QD when dressing the vibrational modes of DNA molecules.

We consider a system composed of a biological semiconductor neutral quantum dot and DNA molecules in the simultaneous presence of a strong control field and a weak signal field. The physical situation is illustrated in Figure 1a. Figure 1b shows the energy levels of peptide QD when dressing the vibrational modes of DNA molecules. The energy levels of peptide quantum dot can be modeled as traditional quantum dot, which consists of two energy states, the ground state |*g*〉 and the first excited state (single exciton) |*ex*〉. Usually, this two-level exciton can be characterized by the pseudospin -1*/*2, operators *S*^± ^and *S*^*z*^. The coherent optical spectroscopy of a strongly driven quantum dot without DNA molecules has been experimentally investigated by Xu et al. [8,9]. Then the Hamiltonian of exciton in a QD can be described by

(1) $$H_{QD}=\hbar \omega_{ex}S^{z},$$

where *ω*_*ex *_is the exciton frequency of peptide quantum dot.

We use the following Hamiltonian to describe DNA molecules, which is modeled as a harmonic oscillator and described by the position and momentum operators *q *and *p*, which have a commutation relation [*q, p*] = *i*ħ [21], and then

(2) $$H_{D}=\overset{n}{\underset{i=1}{\sum }}\left(\frac{p_{i}^{2}}{2m_{i}}+\frac{1}{2}m_{i}\omega_{i}^{2}q_{i}^{2}\right),$$

where *m*_*i *_and *ω*_*i *_are the mass and vibrational frequency of DNA molecule, respectively.

In the unlimited or large volume of aqueous solution, all the vibrational modes of DNA molecules are strongly attenuated. But in a small volume of aqueous solution, the DNA longitudinal vibrational modes decayed slowly, while the other vibrational modes remain strong attenuation [22]. Therefore, for small volume aqueous solution of DNA molecules coupled to a quantum dot system, only the longitudinal modes of DNA molecules can be taken into account. Such vibrational mode of DNA can be simulated as harmonic vibration of spring oscillator. Since the flexion of DNA molecules induces extensions and compressions in the structure of Figure 1a, the longitudinal strain will modify the energy of the electronic states of QD through deformation potential coupling [23,24]. Then the Hamiltonian of the vibrational modes of DNA molecules coupled to the peptide QD can be described by

(3) $$H_{QD-D}=\hbar S^{z}\overset{n}{\underset{i=1}{\sum }}M_{i}q_{i}.$$

where *M*_*i *_is the coupling strength between the peptide QD and the *i*th DNA. It is should be noted that due to the dilute aqueous solution of DNA molecules, here we do not consider the effect of the coupling between the DNA molecules although it may be significant in the dense aqueous solutions [22].

The Hamiltonian of the peptide quantum dot coupled to the strong control field and weak signal field is described as

(4) $$H_{QD-f}=-\mu \left(E_{c}S^{+}e^{-i\omega_{c}t}+E_{c}^{*}S^{-}e^{i\omega_{c}t}\right)-\mu \left(E_{s}S^{+}e^{-i\omega_{s}t}+E_{s}^{*}S^{-}e^{i\omega_{s}t}\right),$$

where *μ *is the electric dipole moment of the exciton, *ω*_*c *_(*ω*_*s*_) is the frequency of the control field (signal field), and *E*_*c *_(*E*_*s*_) is the slowly varying envelope of the control field (signal field). Therefore, we obtain the total Hamiltonian of the coupled peptide QD-DNA in the presence of two optical fields as follows

(5) $$\begin{array}{ll} H & =H_{QD}+H_{D}+H_{QD-D}+H_{QD-f}=\hbar \omega_{ex}S^{z}+\overset{n}{\underset{i=1}{\sum }}\left(\frac{p_{i}^{2}}{2m_{i}}+\frac{1}{2}m_{i}\omega_{i}^{2}q_{i}^{2}\right)\;\\ & \;+\hbar S^{z}\overset{n}{\underset{i=1}{\sum }}M_{i}q_{i}-\mu \left(E_{c}S^{+}e^{-i\omega_{c}t}+E_{c}^{*}S^{-}e^{-i\omega_{c}t}\right)-\mu \left(E_{s}S^{+}e^{-i\omega_{s}t}+E_{s}^{*}S^{-}e^{i\omega_{s}t}\right).\;\\ & \; \end{array}$$

In a rotating frame at the control field *ω*_*c*_, the total Hamiltonian of the system reads as

(6) $$\begin{array}{ll} H & =\hbar \Delta_{c}S^{z}+\overset{n}{\underset{i=1}{\sum }}\left(\frac{p_{i}^{2}}{2m_{i}}+\frac{1}{2}m_{i}\omega_{i}^{2}q_{i}^{2}\right)+\hbar S^{z}Q-\hbar \left(\Omega_{c}S^{+}+\Omega_{c}^{*}S^{-}\right)\;\\ & \;\;-\mu \left(E_{s}S^{+}e^{-i\delta t}+E_{S}^{*}S^{-}e^{-i\delta t}\right),\;\\ & \; \end{array}$$

where Δ_*c *_= *ω*_*ex *_- *ω*_*c*_, $Q=\sum_{i=1}^{n}M_{i}q_{i}$, Ω_*c *_is the Rabi frequency of the control field, and *δ *= *ω*_*s *_- *ω*_*c *_is the detuning between the signal field and the control field.

Furthermore, we may consider the decoherence and relaxation of exciton and DNA mode in combination with their interaction to external environments into the Hamiltonian [25-28]. In general, the environments can be described as independent ensembles of harmonic oscillators with spectral densities. We also assume that DNA molecules interact bilinearly with external environment via its position, and the exciton interacts with the environment through *S*^*x *^operator and *S*^*z *^operator. The *S*^*x *^coupling to the environment models the relaxation process of the exciton, while the *S*^*z *^coupling to the environment models the pure dephasing process of the exciton [25-28]. On the other hand, because *ω*_*ex *_is much larger than *ω*_*i*_, it is reasonable to use the rotating-wave approximation to the exciton-environment coupling term, but not to the DNA-environment coupling term in the system-environment coupling Hamiltonian.

In accordance with standard procedure [25-28], we can obtain the Born-Markovian master equation of the reduced density matrix of the coupled system, *ρ *(*t*), through tracing out the environmental degrees of freedom as

(7) $$\begin{array}{ll} \frac{d\rho }{dt} & =-\frac{i}{\hbar }\left[H,\rho \right]+A\left\{\left[S^{-},\left[S^{+},\rho \right]\right]+h.c.\right\}+B\left\{\left[S^{-},\left\{S^{+},\rho \right\}\right]+h.c.\right\}\;\\ & \;\;+E\left[S^{z},\left[S^{z},\rho \right]\right]+D\left[Q,\left[Q,\rho \right]\right]+G\left[Q,\left[p,p\right]\right]+\frac{i}{\hbar }L\left[Q,\left\{p,\rho \right\}\right],\;\\ & \; \end{array}$$

where the coefficients *A, B, E, D, G*, and *L *correspond to the characteristics of the coupling, and to the structure and properties of the environments. Their explicit form can be written as $A=-\frac{1}{2\hbar }\left\{\frac{\gamma_{1}}{2}\left(1+2N\left(\omega_{ex}\right)\right)\right\}$, $B=\frac{1}{2\hbar }\left\{\frac{\gamma_{1}}{2}\right\}$, $E=-\frac{1}{\hbar }\left\{\frac{\gamma_{2}}{2}\left(1+2N\left(0\right)\right)\right\}$, $D=-\frac{1}{4\hbar }\gamma_{3}\left(1+2N\left(\omega \right)\right)$, $G=-\sum_{i=1}^{n}\frac{\Delta_{3}}{2\hbar m_{i}\omega_{i}}$, $L=-\sum_{i=1}^{n}\frac{\gamma_{3}}{4m_{i}\omega_{i}}$, where *γ*_1 _= 2*π J*_*x *_( ω_*ex*_), *γ*_2 _= 2*π J*_*z*_(0), *γ*_3 _= 2_*π *_*J*_*c*_(*ω*_*i*_) $\Delta_{3}=P\int_{0}^{\infty }d\omega \frac{J_{c}\left(\omega \right)}{\omega -\omega_{i}}\left(1+2N\left(\omega \right)\right)$. $N\left(\omega \right)=\sum_{i=1}^{n}1/\left[\text{exp}\left(\hbar \omega /k_{B}T\right)-1\right]$ is the Boltzman-Einstein distribution of the thermal equilibrium environments. *J*_*x*_, *J*_*z*_, and *J*_*c *_describe the spectral densities of the respective environments coupled through *S*^*x *^and *S*^*z *^to the exciton, and through *Q *to the DNA molecule, respectively. P denotes the principal value of the argument.

According to the master equation (7), we can obtain the equation of motion for the expectation value of any physical operator *O *of the coupled system by calculating $\left\langle Ȯ\left(t\right)\right\rangle =\text{Tr}\left[O\overset{\cdot }{\rho }\left(t\right)\right]$. We thus have

(8) $$\begin{array}{ll} \frac{d}{dt}\left\langle S^{z}\right\rangle & =-\Gamma_{1}\left(\left\langle S^{z}\right\rangle +\frac{1}{2}\right)+i\Omega_{c}\left(\left\langle S^{+}\right\rangle -\left\langle S^{-}\right\rangle \right)\;\\ & \;\;+\frac{i\mu E_{s}e^{-i\delta t}}{\hbar }\left\langle S^{+}\right\rangle -\frac{i\mu E_{s}^{*}e^{i\delta t}}{\hbar }\left\langle S^{-}\right\rangle ,\;\\ & \; \end{array}$$

(9) $$\frac{d}{dt}\left\langle S^{-}\right\rangle =-\left[i\left(\Delta_{c}+Q\right)+\Gamma_{2}\right]\left\langle S^{-}\right\rangle -2i\Omega_{c}\left\langle S^{z}\right\rangle -\frac{2i\mu E_{s}e^{-i\delta t}}{\hbar }\left\langle S^{z}\right\rangle ,$$

(10) $$\frac{d^{2}}{dt^{2}}\left\langle Q\right\rangle +\frac{1}{\tau_{D}}\frac{d}{dt}\left\langle Q\right\rangle +\omega_{D}^{2}\left\langle Q\right\rangle =-\lambda \omega_{D}^{2}S^{z},$$

where $\lambda =\sum_{i=1}^{n}\hbar M_{i}^{2}/\left(m_{i}\omega_{D}^{2}\right)$ corresponds to the peptide QD-DNA coupling strength, *ω*_*D *_is the DNA longitudinal vibrational modes, Γ_1 _and Γ_2 _are the exciton relaxation rate and dephasing rate, respectively, *τ*_*D *_is the vibrational lifetime of DNA molecule [29]. They are derived microscopically as

(11) $$\Gamma_{1}=\frac{2}{\hbar }\left\{\frac{\gamma_{1}}{2}\left(1+2N\left(\omega_{ex}\right)\right)\right\},$$

(12) $$\Gamma_{2}=\frac{1}{\hbar }\left\{\frac{\gamma_{1}}{2}\left(1+2N\left(\omega_{ex}\right)\right)\right\}+\frac{4}{\hbar }\left\{\frac{\gamma_{2}}{2}\left(1+2N\left(0\right)\right)\right\},$$

(13) $$\tau_{D}=\overset{n}{\underset{i=1}{\sum }}\frac{2m_{i}\omega_{i}}{\gamma_{3}}.$$

Note that if the pure dephasing coupling is neglected, i.e., *γ*_2 _= 0, then Γ_1 _= 2Γ_2_. In order to solve these equations, we first take the semiclassical approach by factorizing the DNA molecule and exciton degrees of freedom, i.e., 〈*QS*^*z*^〉 = 〈Q〉〈*S*^*z*^〉, in which any entanglement between these systems should be ignored. And then we make the following ansatz [30]

(14) $$\left\langle S^{-}\left(t\right)\right\rangle =S_{0}+S_{+}e^{-i\delta t}+S_{-}e^{i\delta t},$$

(15) $$\left\langle S^{z}\left(t\right)\right\rangle =S_{0}^{z}+S_{+}^{z}e^{-i\delta t}+S_{-}^{z}e^{i\delta t},$$

(16) $$\left\langle Q\left(t\right)\right\rangle =Q_{0}+Q_{+}e^{-i\delta t}+Q_{-}e^{i\delta t}.$$

Upon substituting these equations to Equations (8)-(10) and working to the lowest order in *E*_*s*_, but to all orders in *E*_*c*_, we finally obtain the linear optical susceptibility *S*_*+ *_in the steady state as the following solution

(17) $$\chi {\left(\omega_{s}\right)}_{eff}^{\left(1\right)}=\frac{\mu S_{+}}{E_{s}}=\frac{\mu^{2}}{\hbar {\text{Γ}}_{2}}\chi (\omega_{s}),$$

where the dimensionless susceptibility *χ*(*ω*_*s*_) is given by

(18) $$\begin{array}{l} \chi (\omega_{s})=\frac{w_{0}}{f(\delta_{0})}\times \{e_{1}e_{2}[2i+\delta_{0})(e_{1}+\delta_{0})-2{\text{Ω}}_{co}^{2}]-e_{2}{\text{Ω}}_{co}^{2}\lambda_{0}\eta w_{0}\\ \;\;\;\;\;\;+{\text{Ω}}_{co}^{2}(2e_{2}-\lambda_{0}\eta w_{0})(e_{1}+\delta_{0})\}, \end{array}$$

where the function *f*(*δ*_0_) and auxiliary function *η*(*ω*_*s*_) are given by

(19) $$\begin{array}{c} f(\delta_{0})=(e_{2}+\delta_{0})\{e_{1}e_{2}[(2i+\delta_{0})(e_{1}+\delta_{0})-2{\text{Ω}}_{co}^{2}]-e_{2}{\text{Ω}}_{co}^{2}\lambda_{0}\eta w_{0}\}\\ \;\;-e_{1}\Omega_{co}^{2}(2e_{2}-\lambda_{0}\eta w_{0})e_{1}+\delta_{0}), \end{array}$$

(20) $$\eta \left(\omega_{s}\right)=\frac{\omega_{D0}^{2}}{\omega_{D0}^{2}-\delta_{0}^{2}-i\delta_{0}/\tau_{D0}},$$

where *e*_1 _= *i *+ Δ_*c*0_-λ_0_/(2*w*_0_), *e*_2 _= *i*-Δ_*c*0 _+ λ_0_/(2*w*_0_), *δ*_0 _= *δ*/Γ_2_, Ω_*c*0 _= Ω_*c*_/Γ_2_, λ_0 _= λ/Γ_2 _ω_*D*0 _= *ω*_*D*_/Γ_2_, *τ*_*D*0 _= τ_*D*_Γ_2_, Δ_*c*0 _= Δ_*c*_/Γ_2_, $w_{0}=2S_{0}^{z}$, and Γ_1 _= 2Γ_2_.

And the population inversion (*w*_0_) of the exciton is determined by the following equation

(21) $$\left(w_{0}+1\right)\left[{\left(\Delta_{c0}-\frac{\lambda_{0}w_{0}}{2}\right)}^{2}+1\right]+2\Omega_{c0}^{2}w_{0}=0.$$

## 3 Results and discussions

For illustration of the numerical results, we choose the realistic coupled system of a peptide QD linked to the DNA molecules in the simultaneous presence of a strong control beam and a weak signal beam as shown in Figure 1. In such coupled system, many DNA molecules linked with one QD. These DNA molecules in solution form may be distorted in mess, but one can extend these molecules into linear form by applying electromagnetic field or fluid force [31]. In addition, the longitudinal vibrational frequency can be determined by the length of DNA molecules. In the theoretical calculation, we select the vibrational frequency and the lifetime of DNA molecule are *ω*_*D *_= 32 GHz and *τ*_*D *_= 3 ns, respectively [24,32-34]. The decay time of peptide quantum dot is 6 *fs *[15], which corresponds to Γ_1 _= 160 THz.

**Figure 2:**
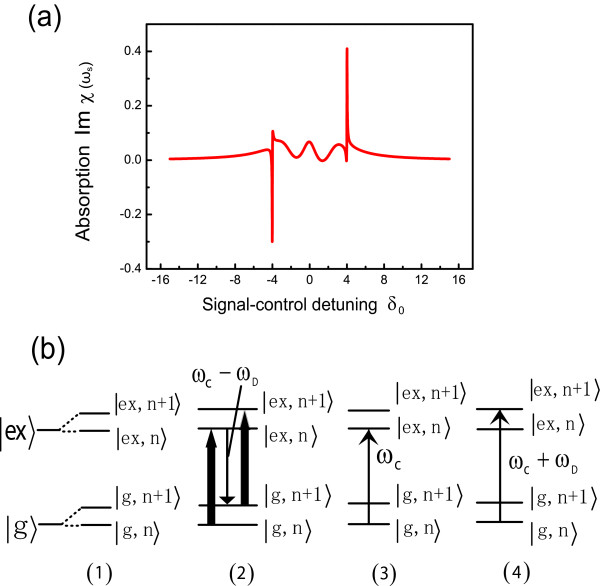
**The absorption spectrum of signal laser and the energy levels of exciton when a single peptide QD coupling with the vibration of DNA molecules**. **(a) **The absorption spectrum of signal laser in the presence of two optical fields for the case $\Omega_{c0}^{2}=2$, Δ_*c*0 _= 0, λ_0 _= 0.5, *ω*_*D*0 _= 4, *τ*_*D*_0 = 33. **(b) **The energy levels of exciton when coupling with the vibration of DNA molecules. Part (2), (3), and (4) correspond to the transitions of the left peak, center feature and right peak, respectively, shown in (a).

Figure 2a shows the signal absorption spectrum as a function of control-signal detuning. In the middle of the figure, we can see that the features appear in the signal absorption spectrum as those in atomic two-level systems [30]. However, the new features which are different from those in atomic systems without DNA molecules also appear at the both sides of the spectrum. Figure 2b gives the origin of these new features. The leftmost (1) of Figure 2b shows the dressed states of exciton when coupling with DNA molecules (|*n*〉 denotes the number states of the DNA molecules). Part (2) shows the origin of DNA vibrational mode induced three-photon resonance. Here the electron makes a transition from the lowest dressed level |*g, n*〉 to the highest dressed level |*ex, n *+ 1〉 by the simultaneous absorption of two control photons and emission of a photon at *ω*_*c *_- *ω*_*D*_. This process can amplify a wave at *δ *= -*ω*_*D*_, as indicated by the region of negative absorption in Figure 2a. The part (3) in Figure 2b shows the origin of DNA stimulated Rayleigh resonance. The Rayleigh resonance corresponds to a transition from the lowest dressed level |*g, n*〉 to the dressed level |*ex, n*〉. Each of these transitions is centered on the frequency of the control laser. The rightmost part (4) corresponds to the usual absorption resonance as modified by the ac Stark effect.

### 3.1 Vibrational frequency measurement of DNA molecule

**Figure 3:**
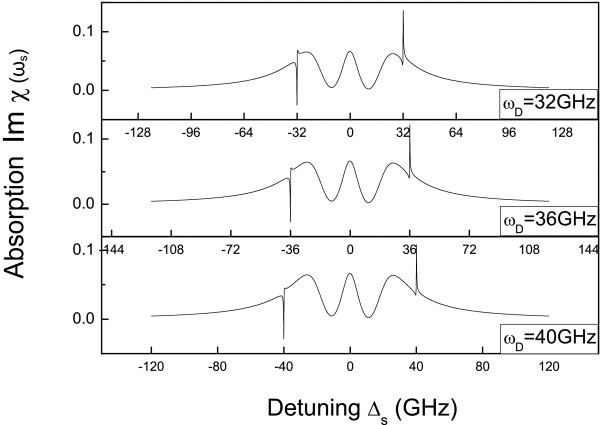
**The absorption spectrum of signal laser with different frequencies of DNA molecule**. The parameters used are *I*_c _= 1.7W/cm^2^, Δ_*c *_= 0, *λ *= 0.8 GHz, *τ*_*D *_= 3 ns.

**Figure 4:**
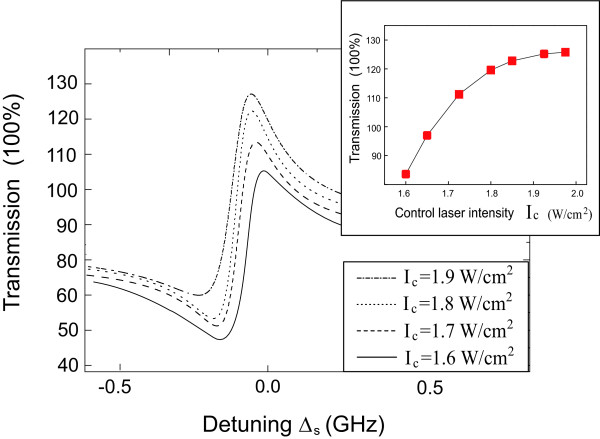
**The characteristic curve of peptide QD-DNA system by plotting the transmission of the signal laser as a function of the control laser intensity**. The other parameters used are *I*_*c *_= 1.7W/cm^2^, Δ_*c *_= -32 GHz, *λ *= 0.8 GHz, *ω*_*D *_= 32 GHz, *τ*_*D *_= 3 ns.

We first fix the control-exciton detuning Δ_*c *_= 0, and scan the signal laser across the exciton frequency the signal spectrum shown in Figure 3 provides us a simple method to detect the frequency of DNA molecule. The two steep peaks shown in the both sides of the spectrum corresponds the vibrational frequency of DNA molecule. For example, if the frequency of DNA is 32 GHz, the two steep peaks will be located at ±32 GHz (the top curve of Figure 3). The location of two peaks can be changed with different frequencies of DNA molecules (the other two plots in Figure 3). In this case, this control-signal technique offered a simple and effective method for the detection of DNA vibrational frequency. We note that the left peak is a negative absorption, which means the output signal light can be amplified in this region. Using the gain region, we may use the peptide QD as a label to discriminate the biological molecules by applying two optical lasers. For more specific description of DNA enhanced spectrum, we further investigate the signal transmission spectrum as a function of the control laser intensity as shown in Figure 4. The amplification of signal laser increases with increasing of control laser intensity as shown in the inset of Figure 4.

### 3.2 Coupling strength determination between peptide quantum dot and DNA molecule

**Figure 5:**
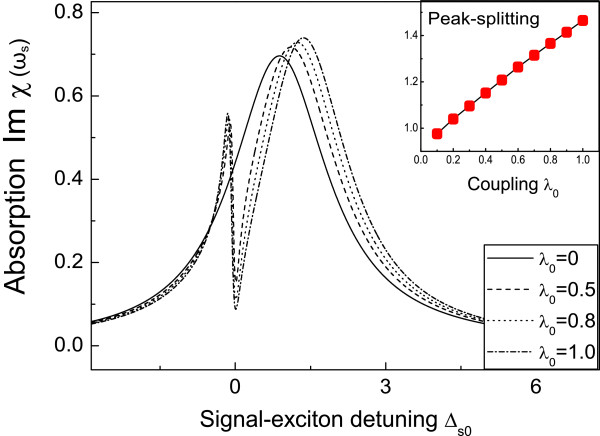
**The absorption spectrum of signal laser as a function of the detuning between signal laser and exciton for four difference coupling of peptide QD-DNA**. The parameters used are $\Omega_{c0}^{2}=2$, Δ_*c*0 _= 4, *ω*_*D*0 _= 4, *t*_*D*0 _= 33.

Next, the coupling strength between peptide quantum dot and DNA molecule can be measured using the pump-probe technique. The absorption spectrum will spit into two peaks and have a zero absorption at Δ_*s *_= 0, if we fix the control laser detuning on the vibrational frequency of DNA molecule (Δ_*c *_= *ω*_*D*_). Figure 5 exhibits that the coupling strength can be measured by the distance of peak splitting in the signal absorption spectrum. However, in the absence of the coupling between peptide quantum dot and DNA molecule, the splitting peaks disappear quickly and turn to a totally absorption peak (see the solid line). This is due to DNA vibration induced coherent population oscillation which makes a deep hole at Δ_*s *_= 0 in the signal absorption spectrum as *δ *= *ω*_*D*_. The inset of Figure 5 shows the linear relationship between the peak splitting and the coupling strength of peptide QD-DNA, which provides us an effective method to detect the coupling strength between QD and DNA. This peak splitting is very similar to the Rabi splitting of two-level systems in quantum optics [35].

Furthermore, in conventional QD-linked biomedicine sensors, excited by single optical field, the fluorescence emission efficiencies still remain challenge due to the coated chemicals, the autofluorescence of background and the copy number of the target to each QD [36]. However, the emission efficiency would be largely enhanced in coherent optical driven by double optical fields, described in this article. From Figure 4, we find that the amplified signal field comes from the quantum interference between the hybrid components and external lasers, which has no relevant with spontaneous fluorescence lifetime of quantum dot. In this case, we anticipate that DNA-linked peptide QD system excited by control-signal technique can be applied to biological imaging, which are nontoxic to environment and human body. For example, once the peptide quantum dot is attached to abnormal DNA molecules, we first apply a strong control field to the peptide QD, provided by Δ_*c *_= -*ω*_D_, then the hybrid system is transparent to other optical fields. Thereafter, we apply a second weak signal beam across the exciton frequency, then the output signal beam can be amplified at Δ_*s *_= 0, which means the peptide quantum dot can be luminant in all-optical domain. Meanwhile, different vibrational frequencies of DNA molecule and the coupling strengths between quantum dot and DNA result in different amplitudes of amplification, which are shown in Figures 4 and 5. This is the DNA enhanced signal spectroscopy of peptide quantum dot, which will have a potential applications in cellular imaging, immunoassays, and clinical diagnosis.

## 4 Conclusions

In this article, we theoretically investigated the coherent optical spectroscopy in a coupled DNA-peptide quantum dot system in the presence of two optical fields. Theoretical analysis shows that the vibrational frequency of DNA and the coupling strength between peptide QD and DNA can be measured effectively and precisely in all-optical domain. Finally, we hope that our predictions in the present study can be testified by experiments in the near future.

## Competing interests

The authors declare that they have no competing interests.

## Authors' contributions

JJL finished the main work of this article, including deducing the formulas, plotting the figures, and drafting the manuscript. KDZ conceived of the idea, participated in its writing and provided some useful suggestions. All authors read and approved the final manuscript.
